# Screening *ANLN* and *ASPM* as bladder urothelial carcinoma-related biomarkers based on weighted gene co-expression network analysis

**DOI:** 10.3389/fgene.2023.1107625

**Published:** 2023-03-27

**Authors:** Tenghao Yang, Zepai Chi, Guoyuan Liu, Xuwei Hong, Sizhe Cao, Kequan Cheng, Yonghai Zhang

**Affiliations:** Department of Urology, Shantou Central Hospital, Shantou, China

**Keywords:** *ANLN*, *ASPM*, bladder urothelial carcinoma, weighted gene co-expression network analysis, WGCNA

## Abstract

**Introduction:** Bladder cancer (BLCA) is one of the most common malignancies in the urinary system with a poor prognosis and high treatment costs. Identifying potential prognostic biomarkers is significant for exploring new therapeutic and predictive targets of BLCA.

**Methods:** In this study, we screened differentially expressed genes using the GSE37815 dataset. We then performed a weighted gene co‐expression network analysis (WGCNA) to identify the genes correlated with the histologic grade and T stage of BLCA using the GSE32548 dataset. Subsequently, Kaplan Meier survival analysis and Cox regression were used to further identify prognosis‐related hub genes using the datasets GSE13507 and TCGA‐BLCA. Moreover, we detected the expression of the hub genes in 35 paired samples, including BLCA and paracancerous tissue, from the Shantou Central Hospital by qRT‐polymerase chain reaction.

**Results:** This study showed that Anillin (ANLN) and Abnormal spindle-like microcephaly-associated gene (ASPM) were prognostic biomarkers for BLCA. High expression of ANLN and ASPM was associated with poor overall survival.The qRT‐PCR results revealed that *ANLN* and *ASPM* genes were upregulated in BLCA, and there was a correlation between the expression of *ANLN* and *ASPM* in cancer tissues and paracancerous tissue. Additionally, the increasing multiples in the *ANLN* gene was obvious in high-grade BLCA.

**Discussion:** In summary, this preliminary exploration indicated a correlation between *ANLN* and *ASPM* expression. These two genes, serving as the risk factors for BLCA progression, might be promising targets to improve the occurrence and progression of BLCA.

## 1 Introduction

Bladder cancer (BLCA) is the tenth most common malignant tumor globally, with approximately 570,000 new cases and 210,000 deaths yearly (Sung et al., 2021). Approximately 75% of patients with BLCA are diagnosed with non-muscle-invasive bladder cancer (NMIBC), whose standard therapy is transurethral resection of bladder tumor (TURBT) combined with intravesical instillation of chemotherapy or immunotherapy. However, 40% of these patients progress to muscle-invasive bladder cancer (MIBC) within 5 years (Sylvester et al., 2006; Babjuk et al., 2019). Specifically, patients with T1G3 NMIBC have a worse prognosis, with disease progression rates of 11.4% and 19.8%, and disease-specific mortality rates of 4.8% and 11.3% in 1 and 5 years, respectively (Cambier et al., 2016). In addition, patients with MIBC are often associated with lymph node metastasis or extravesical invasiveness. A large-sample study showed that the 5-year recurrence-free survival (RFS) of patients with MIBC was about 60%, and cancer-specific survival (CSS) was about 65% after radical cystectomy and pelvic lymphadenectomy (Shariat et al., 2006). However, the CSS of patients with MIBC progressing from NMIBC descended to 35%. The progression to MIBC and bladder cancer related death in high-risk NMIBC were relatively early events occurring within 48 months (van den Bosch and Alfred Witjes, 2011). The RFS of BLCA depends on the lymph node involvement and tumor stage. Studies have shown that with the progression of the tumor stage, the RFS continues to decline: the 5-year RFS of patients with pT1, pT2, pT3, and pT4 BLCA was 76%, 74%, 52%, and 36%, respectively (Stein et al., 2001). Unfortunately, this aggressive biological behavior, coupled with limited treatment options, resulted in a median survival time of only 15 months for patients with metastatic BLCA (von der Maase et al., 2005). This meant that the current treatment strategies based on patient imaging and histopathology could no longer meet the needs of the concept of precise treatment of BLCA. Therefore, exploring diagnosis and prognosis biomarkers for BLCA has great clinical significance.

In this study, we utilized the weighted gene co-expression network analysis (WGCNA) to explore gene modules exhibiting a highly correlated with tumor clinicopathological features (Zhang and Horvath, 2005; Langfelder and Horvath, 2008). This algorithm has been applied to analyze gene expression data in yeast and mice (Carlson et al., 2006; Miller et al., 2010), and has since been extended to explore the biological significance of human tumor-related genes, such as finding the molecular signature of subtypes of non-small-cell lung cancer and the prognostic biomarkers of breast cancer and BLCA (Tang et al., 2018; Niemira et al., 2019; Liu et al., 2021a).

Thus, our study aimed to identify prognostic biomarkers of bladder urothelial carcinoma using WGCNA and validate their expression characteristics, thereby providing novel targets for precision medicine in BLCA.

## 2 Materials and methods

### 2.1 Workflow, data pre-processing, and differential analysis

**FIGURE 1:**
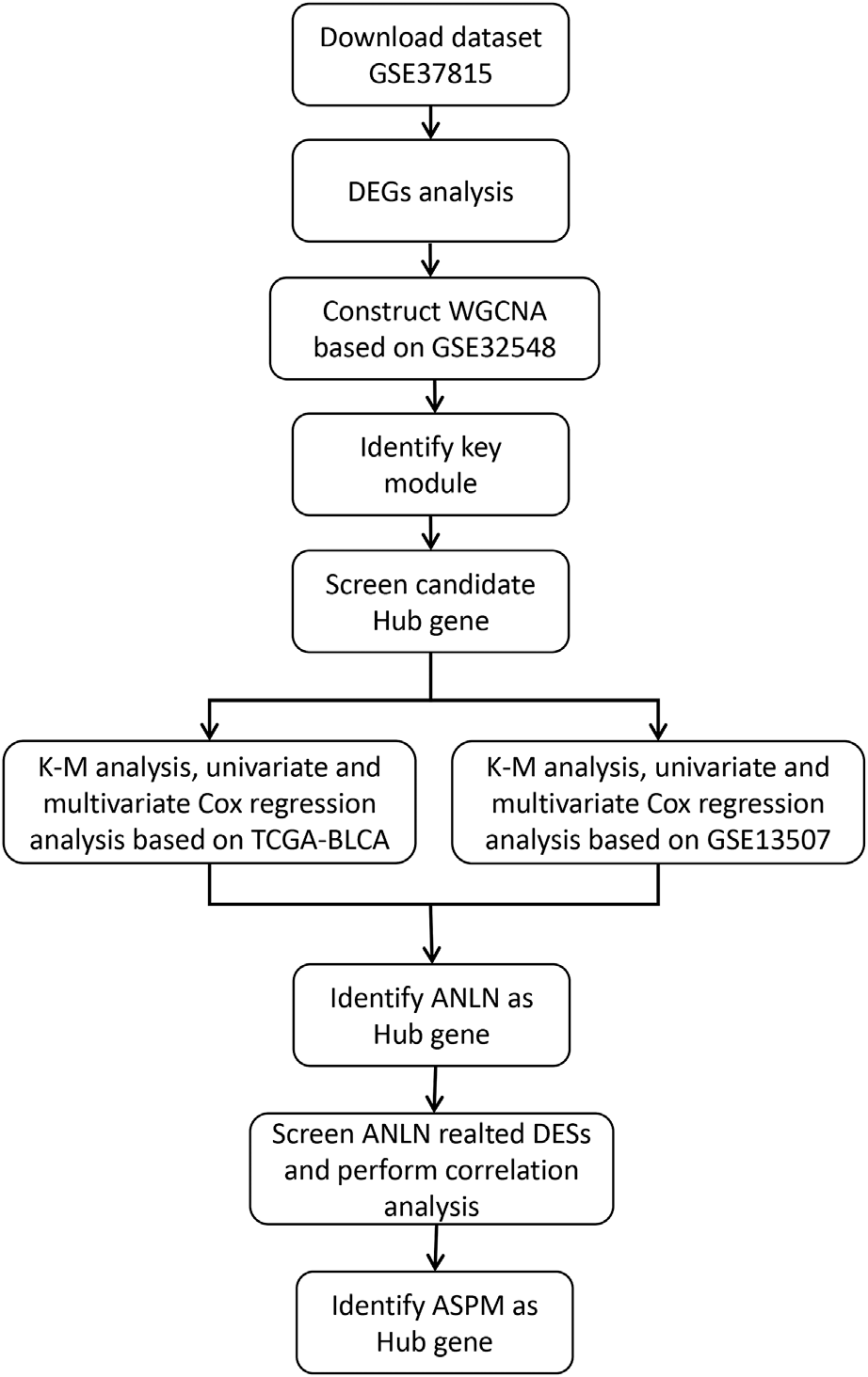
Workflow chart of screening bladder cancer biomarkers based on WGCNA.

The workflow chart is presented in Figure 1. Raw GEO expression data were normalized and log2 transformed using the R package “affy”. The R package “limma” was utilized to screen differentially expressed genes (DEGs) in the dataset GSE37815 which contains 18 BLCA and 6 paracancerous tissue samples. The DEGs screening threshold was set as follows: ① |log2FC| ≥1 ② False discovery rate (FDR) <0.05. Raw TCGA-BLCA dataset were normalized and log2 transformed using the R package “DEseq2”. The dataset GSE32548 was used to construct WGCNA, and the datasets GSE13507 and TCGA-BLCA were used for validation to screen prognostic biomarkers. Samples with missing clinicopathological and survival information were excluded from the aforementioned datasets. The baseline tables of each dataset are presented in Tables 1, 2.

**TABLE 1:** Baseline data sheet of GSE3548 (*N* = 131 cases).

	GSE32548 [*N*(%)]
T Stage	
Ta	40 (30.5)
T1	52 (39.7)
T2	38 (29.0)
TX	1 (0.76)
Grade	
High	15 (11.5)
Median	41 (31.3)
Low	75 (57.3)

**TABLE 2:** Baseline data sheet of TCGA-BLCA (*N* = 400 cases) and GSE13507 (*N* = 165 cases).

	TCGA-BLCA [*N* (%)]	GSE13507[*N* (%)]
Age (year)		
≤65	159 (39.8)	74 (44.8)
>65	241 (60.2)	91 (55.2)
Sex		
Female	103 (25.8)	30 (18.2)
Male	297 (74.2)	135 (81.8)
T Stage		
T1+Ta	1 (1.00)	104 (63.0)
T2	118 (29.5)	31 (18.8)
T3	190 (47.5)	19 (11.5)
T4	56 (14.0)	11 (6.67)
TX	32 (8.00)	—
N Stage		
N0	233 (59.0)	150 (90.9)
N1	44 (11.1)	15 (9.09)
N2	75 (19.0)	
N3	7 (1.77)	
NX	36 (9.11)	—
M Stage		
M0	194 (48.7)	158 (95.8)
M1	11 (2.76)	7 (4.24)
MX	193 (48.5)	—
AJCC Stage		
I	2 (0.50)	—
II	128 (32.2)	—
III	137 (34.4)	—
IV	131 (32.9)	—
Grade		
Low	21 (5.29)	105 (63.6)
High	376 (94.7)	60 (36.4)

### 2.2 WGCNA analysis to screen out candidate hub genes

The R package “WGCNA” was used to construct the co-expression network after matching DEGs with dataset GSE32548. First, we checked all samples and deleted the outliers in dataset GSE32548. Then, we selected an appropriate soft-thresholding parameter β to ensure a scale-free network and construct the adjacent matrix. We used the dynamic cut tree method and set a minimum module size (minClusterSize = 30) and medium sensitivity (deepSplit = 2) to identify gene modules with high topological overlap. Furthermore, the obtained modules were combined with the histologic grade and T stage of BLCA to identify biologically meaningful gene modules. The relationship between genes and external clinicopathological features was further quantified by calculating Gene Significance (GS) and Module Membership (MM). We considered the following three criteria as indicators for screening the candidate hub gene: ① MM > 0.8 ② |GS| > 0.5 ③ *p* < 0.05.

### 2.3 Screening prognostic hub genes by Cox regression and K-M survival analyses

We conducted the univariate and multivariate Cox regression analyses by R package “survival” to further search prognostic genes, using datasets GSE13507 and TCGA-BLCA as validation cohorts. We divided all samples from the validation cohorts into high- and low-expression groups based on the median of gene expression. The R packages “survminer” and “survival” were used to calculate overall survival (OS) and generate the Kaplan Meier (K-M) survival curve. Genes with a significant *p*-value (*p* < 0.05) in Cox regression and OS analyses were regarded as the prognosis-related hub genes.

### 2.4 Identification of the highly related hub genes

To explore genes highly related to the above-mentioned prognostic hub gene, the GSE13507 dataset was divided into high- and low-expression groups based on the median of the hub gene expression. Subsequently, we screened the DEGs by the R package “limma” according to the above grouping. The screening criteria were set as follows: ①|log2FC| ≥2 ② false discovery rate (FDR) < 0.05. Additionally, pearson correlation analysis was performed using the “corrplot” package to compare the degree of correlation between genes.

### 2.5 Patients and tissue samples

From August 2020 to December 2021, 35 paired BLCA and paracancerous tissue samples were collected from newly diagnosed patients admitted to the Urology Department of Shantou Central Hospital. All patients underwent TURBT, and their postoperative pathological diagnosis was bladder urothelial carcinoma. BLCA and paracancerous tissue samples were collected during the surgery and stored in a −80°C refrigerator after frozen by liquid nitrogen. Additionally, we recorded patient clinicopathological information, including age, sex, BLCA invasion depth, histologic grade, tumor size, single or multiple tumors, primary or recurrent, and pre-treatment urine routine. The baseline information of 35 patients with BLCA is presented in Table 3. This research project was approved by the Medical Ethics Committee of Shantou Central Hospital.

**TABLE 3:** Baseline table of 35 patients with bladder cancer in the Department of Urology, Shantou Central Hospital.

	*N* (%)	Sum
Sex		35
Male	31 (88.6)	
Female	4 (11.4)	
Age (year)	62.83 ± 13.26	35
Invasion depth		35
MIBC	9 (25.71)	
NMIBC	26 (74.29)	
Grade		35
Low	20 (57.14)	
High	15 (42.86)	
Diameter (cm)	2.75 ± 1.19	35
Number		35
Single	20 (57.14)	
Multiple	15 (42.86)	
Recurrent or not		35
Recurrent	5 (14.29)	
Primary	30 (85.71)	
Urine WBC		35
Negative	17 (48.57)	
Positive	18 (51.43)	
Urine nitrite		35
Negative	31 (88.57)	
Positive	4 (11.43)	
Urine RBC		35
Negative	7 (20.00)	
Positive	28 (80.00)	

### 2.6 RNA extraction and quantitative real-time polymerase chain reaction

Total RNA was extracted from tissue samples using Trizol reagent (Ambion, United States) following the manufacture’s protocol. The concentration and purity of extracted RNA were measured using a spectrophotometer (Allsheng, China). Then, complementary DNA (cDNA) was synthesized by reverse transcription using HiScript Reverse Transcriptase (RNase H) (VAZYME, China). Subsequently, quantitative real-time polymerase chain reaction (qRT-PCR) was performed using SYBR Green Master Mix (VAZYME, China) on an ABI QuantStudio 6 Real-Time PCR System (Applied Biosystems, United States). The relative expressions levels of related genes were detected using the 2^−ΔΔCT^ method. The primer sequences used for amplification were as follows: Anillin (*ANLN*) forward 5′-GTG​ATT​CTG​TTG​CTG​TCC​CG-3′ and reverse 5′-GCA​GCC​TTT​TCC​TCT​GAT​GG-3′ primers; Abnormal spindle-like microcephaly-associated gene (*ASPM*) forward 5′-CGT​CAC​CTT​GGC​TTA​TTG​GG-3′ and reverse 5′-GAG​GTC​CCA​GTT​CTG​TGT​GA-3′ primers; *β-action* forward 5′-AGC​GAG​CAT​CCC​CCA-AAG​TT-3′ and reverse 5′-GGG​CAC​GAA​GGC​TCA​TCA​TT-3′ primers.

### 2.7 Statistical analysis

The R software 3.6.3 and IBM SPSS software 25.0 were used for all statistical analyses. The data were presented as mean ± standard deviation. The Wilcoxon signed-rank test was used to compare the PCR results of tumor and paracancerous tissues. The Mann Witney Wilcoxon test was used to compare gene expression and clinicopathological characteristics, and Pearson’s coefficient was used to examine the correlation between hub genes. K-M survival curves were constructed to analyze OS between the high-expression and the low-expression groups. Cox regression was performed to screen the related risk factors for BLCA prognosis. A *p*-value of less than 0.05 was considered statistically significant.

## 3 Results

### 3.1 Selection of DEGs and candidate hub genes *via* WGCNA

**FIGURE 2:**
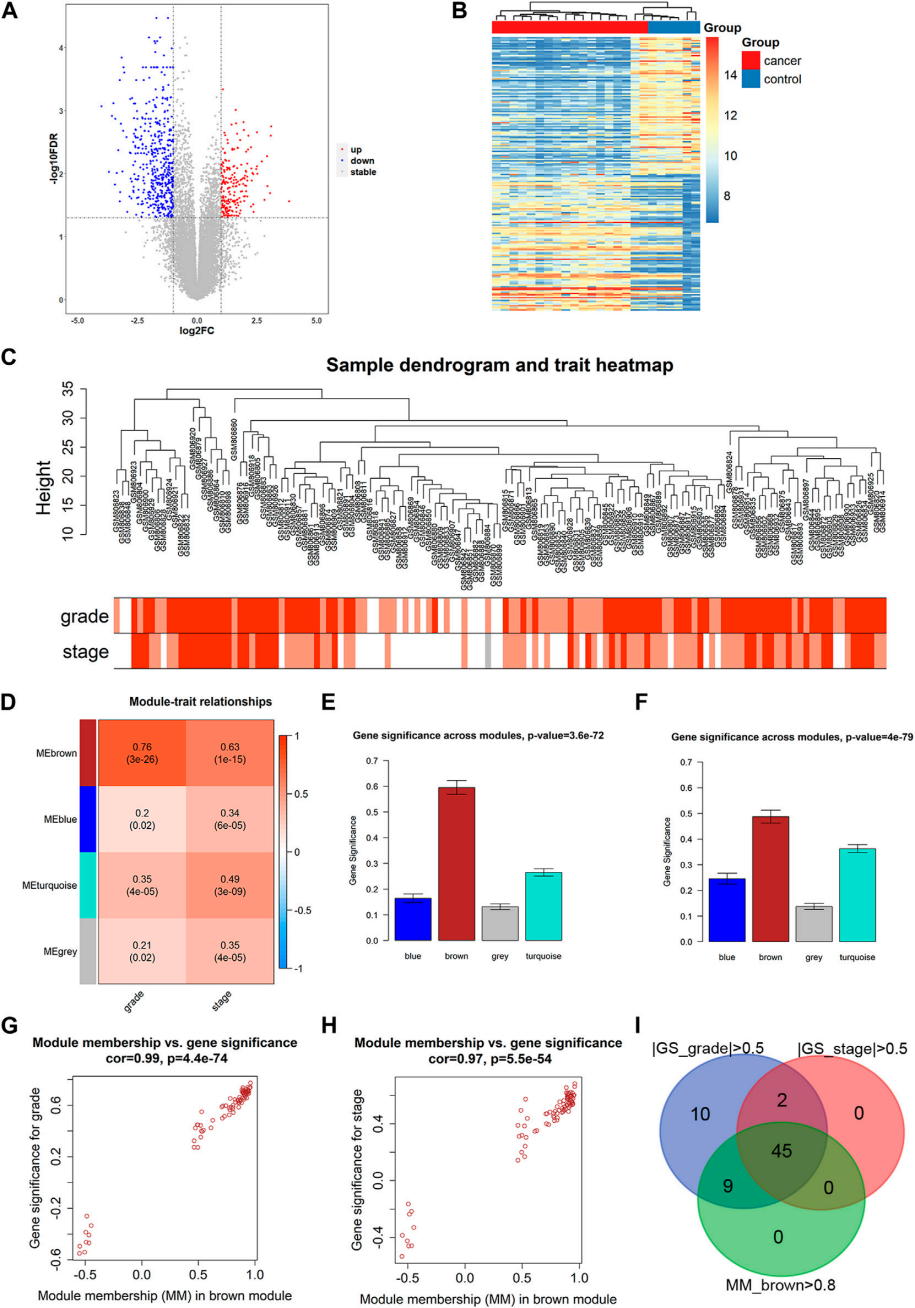
DEG analysis and WGCNA construction. **(A)** Volcano plot of GSE37815 DEGs. **(B)** Heatmap of GSE37815 DEGs. **(C)** GSE32548 clustering results and corresponding grade and stage information. **(D)** Correlation between ME of GSE32548 and clinical sample information. Distribution of GS and errors in the modules associated with the histologic grade **(E)** and T stage **(F)** of bladder cancer. Scatter plot of GS and MM related to the histologic grade **(G)** and T stage **(H)** of bladder cancer in brown module. **(I)** Venn diagram of bladder cancer grading-related GS, staging-related GS and brown module MM. ME, module eigengenes; GS, gene significance; MM, module membership.

Through differential expression analysis, we identified 694 DEGs (207 upregulated and 487 downregulated) in the GSE37815 dataset. The volcano plot and heatmap of DEGs are shown in Figures 2A, B. Then, 694 DEGs were used to construct WGCNA using the GSE32548 dataset. All 131 samples and their clinical information were included in the analysis without removing any outlier samples (Figure 2C). The network was constructed using a soft threshold β = 9 (scale-free R2 = 0.91) (Supplementary Figure S1 and Supplementary Table S1), and we identified four modules: brown, blue, turquoise, and grey, containing 158, 87, 180, and 267 genes, respectively (Supplementary Figure S2). Among these modules, the brown module showed the most significant correlation with histologic grade (cor = 0.76, *p* < 0.01) and T stage (cor = 0.63, *p* < 0.01) and was considered to be the key module in this study (Figure 2D). In addition, we examined the GS and MM related to histologic grade and T stage of BLCA in the brown module, as shown in Figures 2E–H. According to the candidate hub genes screening threshold, we identified 45 genes for further survival analysis (Figure 2I).

### 3.2 Identification of *ANLN* as a prognosisrelated hub gene

**FIGURE 3:**
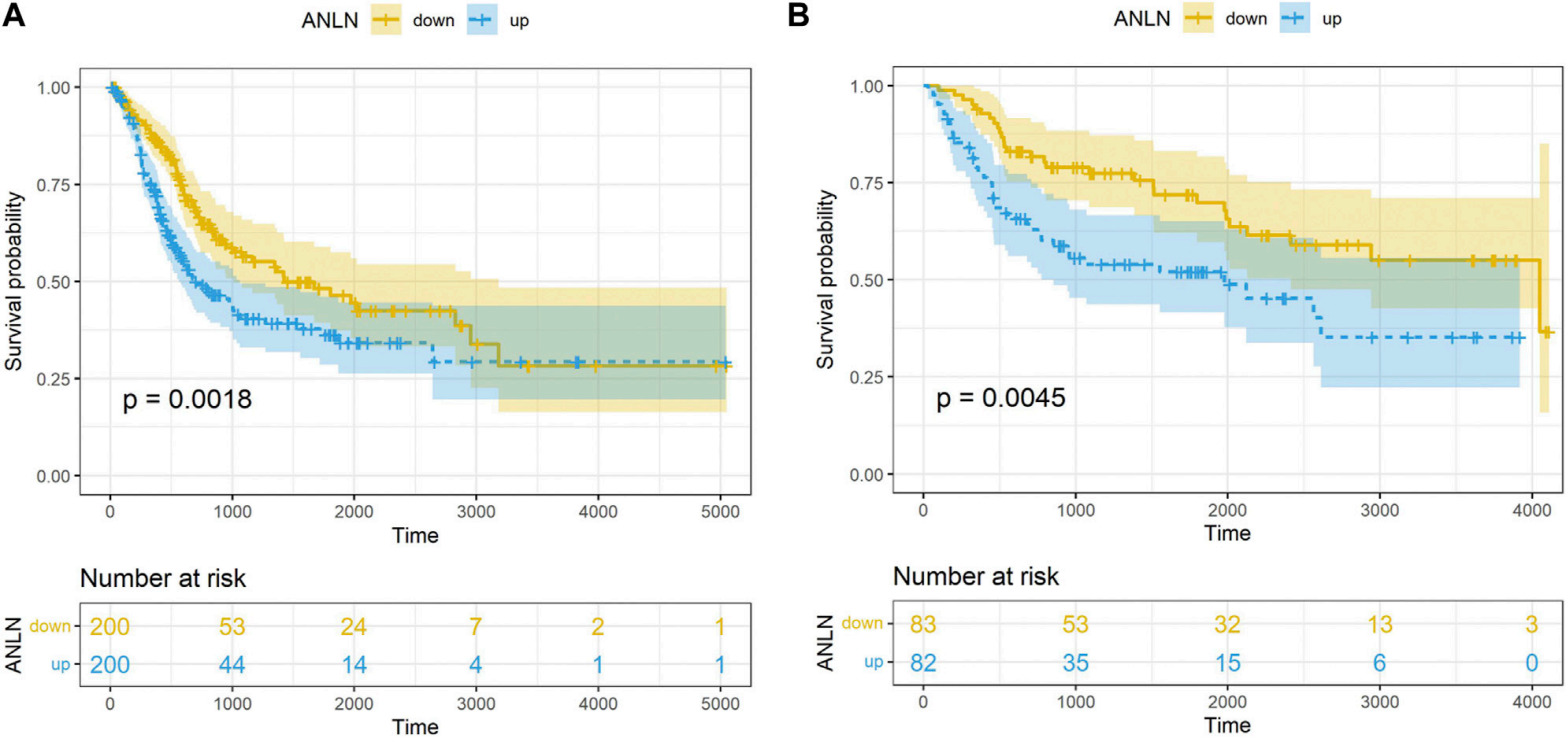
K-M survival analysis on the *ANLN* gene in dataset TCGA-BLCA **(A)**, GSE13507 **(B)**.

The prognostic value of 45 candidate hub genes was validated in this study using the TCGA-BLCA and GSE13507 validation datasets. Firstly, we conducted univariate Cox regression analysis and found that the *ANLN* gene (HR = 1.23, 95% CI = 1.07–1.42, *p* = 0.003) was the only risk factor in the TCGA-BLCA dataset. In the GSE13507 dataset, univariate Cox regression analysis identified 43 genes related to BLCA prognosis, including *ANLN* (HR = 1.47, 95% CI = 1.18–1.83, *p* = 0.001). Subsequently, we performed multivariate Cox regression analysis including the 43 genes and identified *ANLN* (HR = 2.83, 95% CI = 1.29–6.21, *p* = 0.001) as a potential factor affecting the prognosis of BLCA. K-M survival curve suggested that the high-expression of *ANLN* had a poor OS in both datasets TCGA-BLCA and GSE13507 datasets (Figures 3A, B).

### 3.3 Screening of *ASPM* as a highly related gene with *ANLN*

**FIGURE 4:**
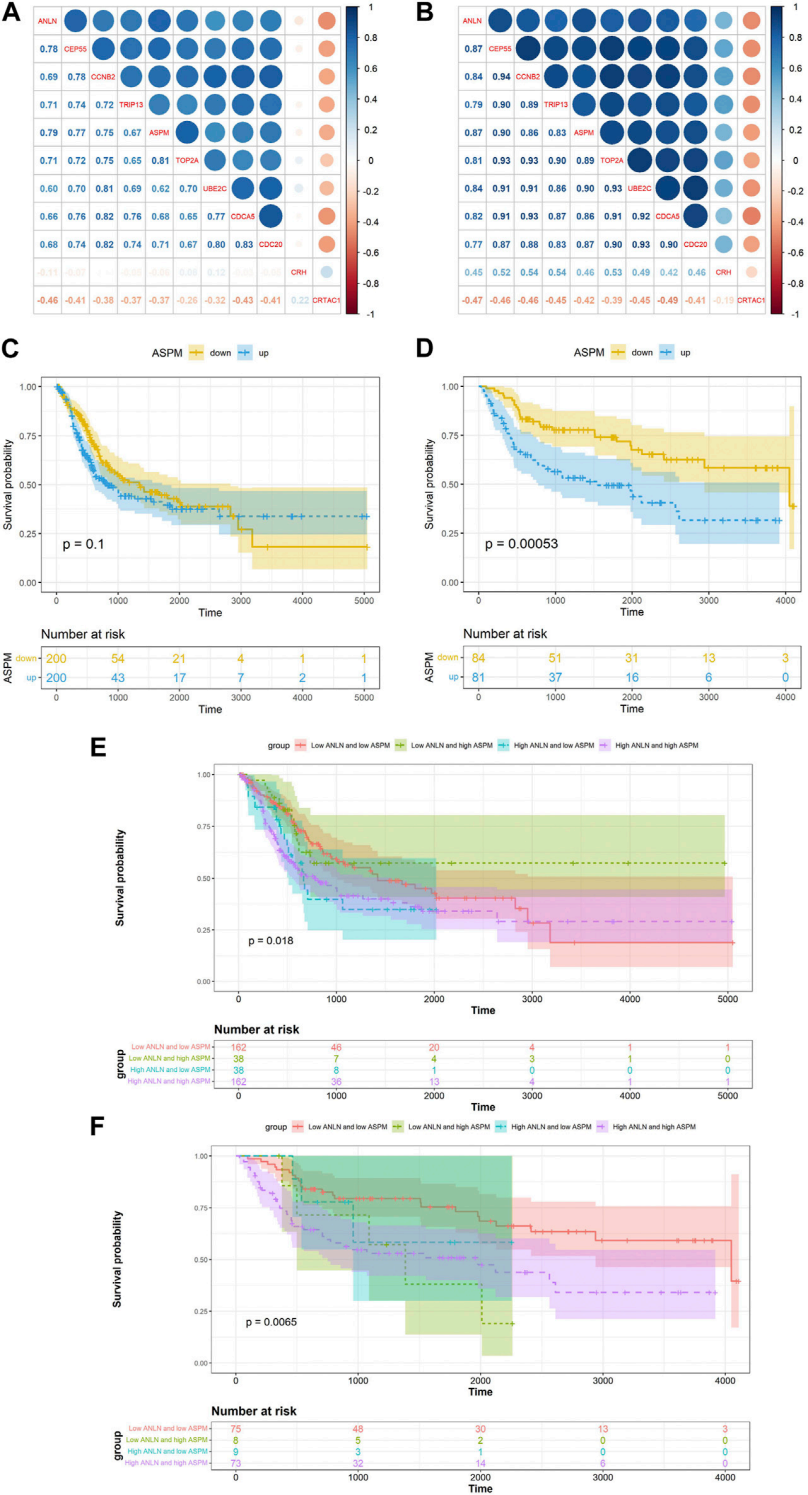
Correlation between *ANLN* related DEGs and *ANLN* based on TCGA-BLCA **(A)** and GSE13507 **(B)**. K-M survival curves of *ASPM* in bladder cancer based on TCGA-BLCA **(C)** and GSE13507 **(D)**. The K-M survival curves of *ANLN* and *ASPM* with different expression levels in bladder cancer based on TCGA-BLCA **(E)** and GSE13507 **(F)**.

After determining *ANLN* as a hub gene, we performed differential expression analysis based on the median of *ANLN* expression in the GSE13507. We identified 10 DEGs (*CEP55, CCNB2, TRIP13, ASPM, TOP2A, UBE2C, CDCA5, CDC20, CRH and CRTAC1*) using the following screening criteria: ① |log2FC| ≥ 2 ② false discovery rate (FDR) < 0.05. Pearson correlation analysis revealed that *ASPM* exhibited the highest correlation with *ANLN* among all 10 DEGs in both TCGA-BLCA (R = 0.79, *p* < 0.01) and GSE13507 (R = 0.87, *p* < 0.01) datasets (Figures 4A, B). Moreover, high expression of *ASPM* was associated with shorter OS in the TCGA-BLCA and GSE13507 datasets, as detected by K-M survival analysis (Figures 4C, D). Meanwhile, we found that high expression of both *ANLN* and *ASPM* was associated with shorter survival time compared to low expression of *ANLN* and *ASPM* in the TCGA-BLCA and GSE13507 datasets (Figures 4E, F).

### 3.4 Expressions of *ANLN* and *ASPM* was correlated and upregulated in bladder cancer

**FIGURE 5:**
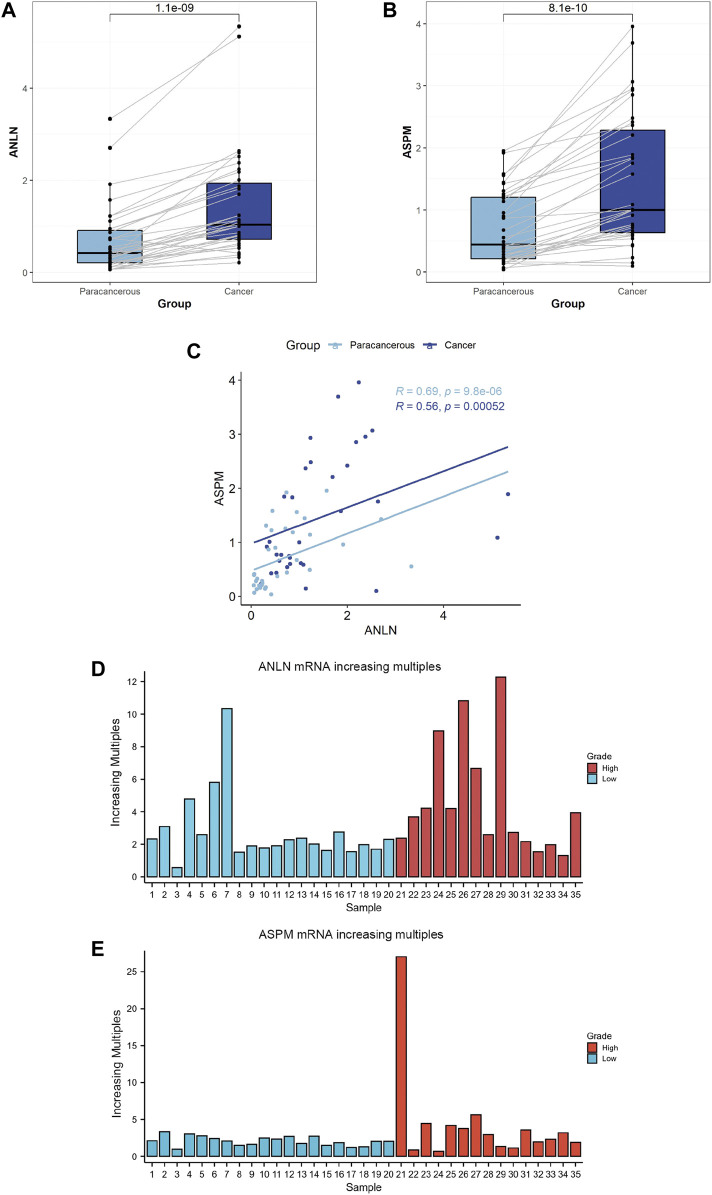
Comparison between bladder cancer tissues (*N* = 35) and paracancerous tissues (*N* = 35) according to the expression levels of *ANLN* **(A)** and *ASPM* genes **(B)**. Correlation between *ANLN* and *ASPM* in bladder cancer tissues (*N* = 35) and paracancerous tissues (*N* = 35) **(C)**. Comparison between bladder cancer tissues and normal tissues according to the increasing multiples in the mRNA expression levels of *ANLN* **(D)** and *ASPM* **(E)**.

We collected paired samples of BLCA and paracancerous tissues, and measured the mRNA expression levels of *ANLN* and *ASPM* using RT-qPCR. As shown in Figures 5A, B and Table 4, the mRNA expression levels of *ANLN* and *ASPM* were significantly upregulated in BLCA. These results were consistent when we stratified patients with BLCA into NMIBC and MIBC. Furthermore, we found that there was a correlation between the expression of *ANLN* and *ASPM* in BLCA (R = 0.56, *p* < 0.01) and paracancerous tissues (R = 0.69, *p* < 0.01), as shown in Figure 5C. To further investigate the relationship between hub genes and BLCA, we defined an increasing multiples as the ratio of mRNA expression levels in BLCA tissues to those in paracancerous tissues. Our results showed that *ANLN* expression was upregulated in BLCA in 34 out of 35 patients (97.1%) (Figure 5D). Additionally, the increasing multiples of *ANLN* were significantly higher in high-grade BLCA than in low-grade (low-grade: 2.26 ± 2.12 vs. high-grade: 4.63 ± 3.47, *p* = 0.046). Similarly, we found that 32 out of 35 patients (91.4%) had higher mRNA expression levels of *ASPM* in BLCA than in paracancerous tissues (Figure 5E). However, there was no significant difference in the increasing scores of *ASPM* between high-grade and low-grade BLCA patients (low-grade: 2.10 ± 0.64 vs. high-grade: 4.34 ± 6.44, *p* = 0.194).

**TABLE 4:** Comparison between bladder cancer tissues and paracancerous tissues according to the expression levels of *ANLN* and *ASPM* genes in total samples (*N* = 35), NMIBC (*N* = 26) and MIBC (*N* = 9).

	Bladder cancer	Paracancerous tissue	*Z*	*p*
Total				
*ANLN* gene expression				
Median (Min-Max)	1.035 (0.210–5.339)	0.421 (0.058–3.332)	5.037	<0.001
Mean ± SD	1.445 ± 1.178	0.673 ± 0.747		
*ASPM* gene expression				
Median (Min-Max)	1.000 (0.097–3.957)	0.439 (0.037–1.953)	5.053	<0.001
Mean ± SD	1.461 ± 1.061	0.706 ± 0.577		
NMIBC				
*ANLN* gene expression				
Median (Min-Max)	1.11 (0.210–5.339)	0.437 (0.058–2.703)	4.445	<0.001
Mean ± SD	1.480 ± 1.082	0.664 ± 0.646		
*ASPM* gene expression				
Median (Min-Max)	1.380 (0.097–3.957)	0.678 (0.068–1.953)	4.368	<0.001
Mean ± SD	1.605 ± 1.148	0.793 ± 0.613		
MIBC				
*ANLN* gene expression				
Median (Min-Max)	0.793 (0.328–5.119)	0.315 (0.061–3.332)	2.369	0.012
Mean ± SD	1.344 ± 1.491	0.700 ± 1.032		
*ASPM* gene expression				
Median (Min-Max)	0.915 (0.425–2.479)	0.396 (0.037–1.309)	2.488	0.009
Mean ± SD	1.047 ± 0.637	0.454 ± 0.377		

Z, Wilcoxon signed-rank test; *p*: *p*-value for comparing bladder tissues and paracancerous tissues.

### 3.5 Association of *ANLN* and *ASPM* with the clinicopathological features of patients with bladder cancer

**FIGURE 6:**
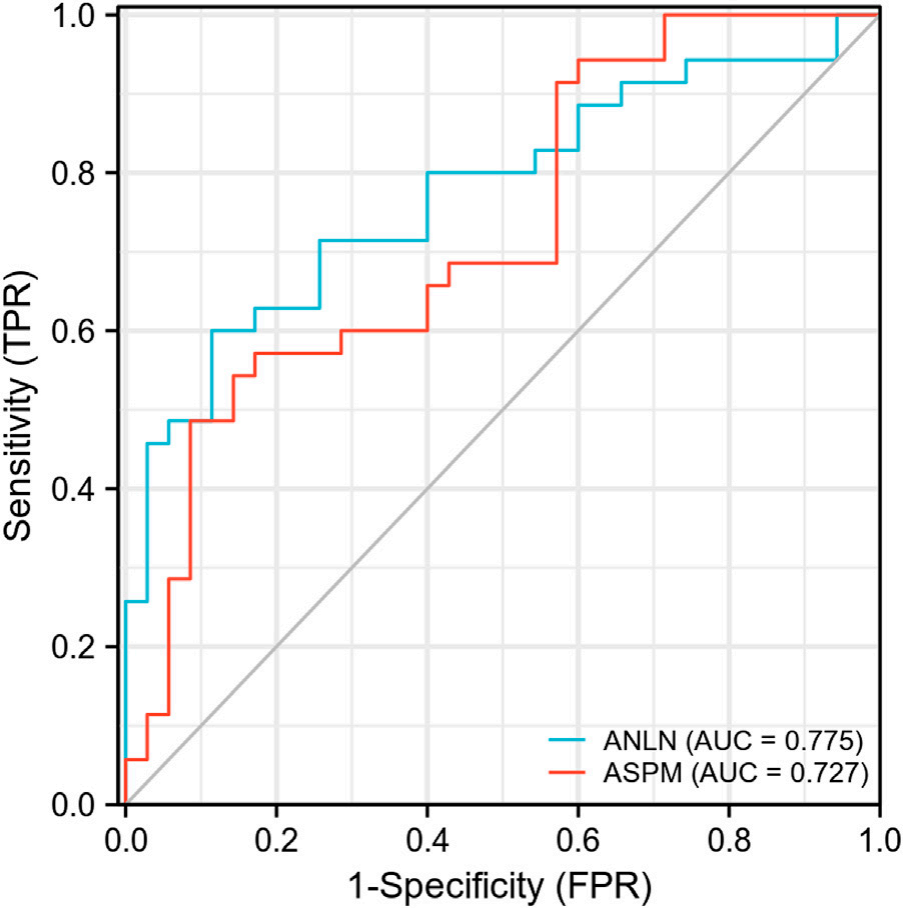
ROC curve for the expression levels of both *ANLN* and *ASPM* genes in bladder cancer tissues *versus* paracancerous tissues. ROC, receiver operating characteristics; AUC, area under the curve.

An ROC curve was performed to evaluate the diagnostic significance based on the mRNA expression levels of both the *ANLN* and *ASPM* genes. The *ANLN* gene exhibited a sensitivity of 88.6% and a specificity of 60% (*p* < 0.01), while the *ASPM* gene had a sensitivity of 82.9% and a specificity of 57.1% (*p* < 0.01) (Figure 6). Furthermore, we analyzed the relationship between the expression of *ANLN* and *ASPM* genes with the clinicopathological characteristics of BLCA. The results suggested no statistically significant difference between the mRNA expression levels of *ANLN* and *ASPM* and different clinicopathological features (Table 5).

**TABLE 5:** Relationship of the expression levels of *ANLN* and *ASPM* genes with clinicopathological characteristics in bladder cancer.

	*N*	*ANLN* ( $\bar{x}\pm s$ )	*ASPM* ( $\bar{x}\pm s$ )
Invasion depth			
NMIBC	26	1.480 ± 1.082	1.604 ± 1.148
MIBC	9	1.345 ± 1.491	1.047 ± 0.636
*Z* (*p*)		−1.095 (0.274)	−1.057 (0.290)
Grade			
Low	20	1.345 ± 1.167	1.726 ± 1.164
High	15	1.579 ± 1.218	1.108 ± 0.813
*Z* (*p*)		−0.867 (0.386)	−1.300 (0.193)
Number			
Single	20	1.426 ± 1.121	1.456 ± 1.162
Multiple	15	1.471 ± 1.289	1.467 ± 0.949
*Z* (*p*)		−0.100 (0.920)	−0.100 (0.920)
Recurrent or not			
Primary	30	1.450 ± 1.232	1.452 ± 1.038
Recurrent	5	1.415 ± 0.880	1.514 ± 1.323
*Z* (*p*)		−0.236 (0.814)	−0.236 (0.814)
Urine WBC			
Negative	16	1.574 ± 1.204	1.730 ± 0.988
Positive	19	1.323 ± 1.173	1.207 ± 1.092
*Z* (*p*)		−0.957 (0.338)	−1.915 (0.056)
Urine RBC			
Negative	7	1.714 ± 1.678	1.393 ± 0.893
Positive	28	1.378 ± 1.047	1.478 ± 1.113
*Z* (*p*)		−0.041 (0.967)	−0.413 (0.680)
Urine nitrite			
Negative	31	1.497 ± 1.243	1.427 ± 1.069
Positive	4	1.045 ± 0.182	1.724 ± 1.103
*Z* (*p*)		−0.052 (0.959)	−0.570 (0.568)

*Z*, Mann–Whitney test; *p*, *p*-value for the association between expression levels of *ANLN* or *ASPM* genes and different parameters.

## 4 Discussion

More than 1.6 million people live with BLCA worldwide. About 1 in 100 men and 1 in 400 women are diagnosed with BLCA sometime in life globally. BLCA has the highest lifetime treatment costs per patient among all cancer types, with an annual cost of 3.6 billion euro in the United States and almost 5 billion euro in Europe (Richters et al., 2020). Urothelial carcinomas account for 90% of bladder malignant tumors histologically (Lobo et al., 2020). While intravesical *Bacillus* Calmette-Guerin instillation therapy remains a crucial option for the patients with intermediate and high-risk NMIBC after TURBT, the research into the molecular biology and genetics of BLCA has revolutionized the treatment of MIBC and advanced disease, including immunotherapy and checkpoint inhibition, targeted therapy, and antibody-drug conjugates (Lenis et al., 2020). Therefore, identifying valuable diagnostic, therapeutic, and prognostic targets for BLCA to achieve personalized and precise treatment of BLCA is of clinical significance.

In this study, we utilized bioinformatics analysis to identify *ANLN* and *ASPM* genes as prognostic biomarkers for BLCA. The upregulation of *ANLN* and *ASPM* genes was observed in BLCA, and their high expression was associated with poor OS in patients with BLCA. The results of RT-qPCR further confirmed the upregulation of ANLN and ASPM in BLCA, which was observed in both MIBC and NMIBC. By comparing the increasing multiples, we found that the increasing multiples of ANLN was higher in high-grade tumors. The proportion of upregulated ANLN and ASPM was 97.1% and 91.4%, respectively, indicating that the upregulation of both genes was a common occurrence in patients with BLCA.

*ANLN* is located on the short arm of human chromosome 7 and encodes a conserved multi-domain protein that interacts with various biological partners, acting as a scaffold to organize the cytoskeleton (Piekny and Glotzer, 2008). ANLN was originally isolated from *Drosophila melanogaster* embryonic extract and could bind to F-actin. Therefore, it was also called Anillin actin-binding protein (Field and Alberts, 1995). During cytokinesis, ANLN interacts with regulators of cytoskeletal components such as actin, septins, RhoA, and Ect2, among others, to facilitate cytoplasmic division, morphological changes, and ultimately, cell division into two subunits (Tuan and Lee, 2020). Notably, the injection of antibodies targeting ANLN has been shown to slow furrow ingression and lead to the failure of cytokinesis (Oegema et al., 2000). Similarly, the depletion of ANLN in human or *Drosophila* cultured cells induces lateral furrow oscillation that fails cytokinesis (Piekny and Maddox, 2010). *ANLN* has been identified as a upregulated gene in various tumors driving cell proliferation (Tuan and Lee, 2020). In non-small cell lung cancer, inhibition of the *ANLN* gene expression could suppress tumor cell growth, resulting in larger morphology, multiple nuclei, and ultimately cell death (Suzuki et al., 2005). Knockdown of the *ANLN* gene in mice hepatocytes blocked cytokinesis and inhibited liver tumor development (Zhang et al., 2018). A study using transcriptome sequencing found that *ANLN* was significantly upregulated in BLCA tissues (log2FC = 2.926) (Zeng et al., 2017). PCR and immunohistochemical experiments on BLCA and paracancerous normal mucosal tissues showed that the expression of the *ANLN* gene is upregulated in BLCA, especially in MIBC and high-grade cancers. Meanwhile, patients with BLCA in the high-*ANLN* expression group showed poor CSS, progression-free survival (PFS) and RFS, compared with the low-*ANLN* expression group (Zeng et al., 2017). Knockdown of the *ANLN* gene could inhibit BLCA cell proliferation, migration, and invasion, as well as induce cell cycle arrest and abnormal binucleation (Zeng et al., 2017; Chen et al., 2022). Patients with MIBC exhibiting high expression of the *ANLN* gene had shorter OS and disease-specific survival after radical cystectomy (Wu et al., 2019). Taken together, these findings suggested that the expression of *ANLN* gene was upregulated in BLCA and promoted the proliferation and invasion of BLCA cells. High expression of *ANLN* predicted poor prognosis, which was consistent with our analysis.

*ASPM* is located on the long arm of human chromosome 1, encoding a large protein of 3,477 amino acids (410 kDa). This protein comprises a putative amino-terminal microtubule-binding domain, two calponin homology domains that commonly involve in actin binding, 81 IQ (isoleucine-glutamine) repeat motifs that potentially bind to calmodulin, and a carboxy-terminus (C-terminal domain) of unknown function (Kouprina et al., 2005; Zhong et al., 2005; Paramasivam et al., 2007). The human *ASPM* gene produces two splice isoforms of 410 and 250 kDa, enriched in centrosomes and intermediates, respectively. This suggests that these isoforms may undertake the significant cellular functions of ASPM and participate in the regulation of mitosis (Paramasivam et al., 2007). The homologous proteins of human *ASPM* in *Drosophila*, *asp*, is involved in spindle microtubule organization and cytokinesis during mitosis and meiosis. Mutations in the human *ASPM* gene can lead to the defective proliferation of neural progenitor cells, resulting in autosomal recessive primary microcephaly (MCPH) (Bond et al., 2002; Roberts et al., 2002; Bond et al., 2003). Studies have shown that ASPM participates in spindle organization, spindle positioning, and cytokinesis in all dividing cells, not just nerve cells, and widely expresses in various fetal and adult tissue cells, including liver, lung, kidney (Kouprina et al., 2005; Higgins et al., 2010). The *ASPM* gene is upregulated in various tumors, including ovarian cancer, breast cancer, colon cancer, glioblastoma, and BLCA (Kouprina et al., 2005; Saleh et al., 2020). Patients with BLCA who have high expression levels of *ASPM* had shorter OS and PFS. The proliferation rate of the T24 cell line transfected with si-*ASPM* was significantly lower than that in the si-Control group (Xu Y. et al., 2020). Nude mice receiving *ASPM* knockdown T24 cells developed smaller tumors with lower tumor proliferative activity, indicating a significant role of *ASPM* in BLCA proliferation and tumorigenesis (Gao et al., 2020). Additionally, the single-cell transcriptome analysis identified a group of ASPM + basal-like cell types, the number of which significantly increased in acute bladder urothelium injury caused by uropathogenic *Escherichia coli*. This suggested that ASPM + urothelial cells were involved in the bladder urothelial regeneration after injury (Li et al., 2021). Furthermore, bioinformatics analysis revealed that high expression of the *ASPM* gene is associated with the poor prognosis of patients with BLCA, indicating that BLCA originating from this ASPM + subpopulation might be highly malignant and invasive (Li et al., 2021).

Spearman correlation analysis revealed a correlation between the mRNA expression of *ANLN* and *ASPM* in BLCA and paracancerous tissues. The correlation was higher in paracancerous tissues, indicating that the interaction between *ANLN* and *ASPM* also exists in normal tissues. Previous studies have confirmed *ANLN* and *ASPM* as risk factors for the prognosis of patients with BLCA through silico data analysis (Chen et al., 2019; Wang et al., 2019; Xu et al., 2019; Li et al., 2020; Liu et al., 2021b). Our study aimed to further discuss the impact of these genes on BLCA. ANLN is a highly conserved multi-domain protein that interacts with multiple cytoskeletal components during cell division (Tuan and Lee, 2020). ASPM participates in regulating microtubules, which are the cytoskeletal fibers involved in various physiological functions of cells (Akhmanova and Steinmetz, 2019). Therefore, both ANLN and ASPM are cytoskeleton-associated proteins involved in cytokinesis (Schiewek et al., 2018). The cytoskeleton plays a crucial role in maintaining the shape and internal organization of cells, as well as in cell division and migration. In particular, cell migration is initiated by an actin-dependent protrusion of the cell’s leading edge, which comprises structures known as lamellipodia and filopodia (Imai-Sumida et al., 2017). Regulating the formation of cytoskeleton and pseudopodia promotes the invasion and metastasis of BLCA (Xu N. et al., 2020). Remodeling of the intracellular cytoskeleton is one of the critical mechanisms of tumorigenesis, as the cytoskeleton directly regulates two major characteristics of cancer cells: proliferation and motility (Jiang et al., 2009; Sagona and Stenmark, 2010; Akhshi et al., 2014; Fife et al., 2014). Human cells contain four major cytoskeletal components: actin, microtubules, intermediate filaments, and septin polymers (Spiliotis and Nelson, 2006; Hohmann and Dehghani, 2019). These components have complex interactions, control cellular organization through signaling crosstalk, and play an essential role in tumor development (Jiang et al., 2009; Sagona and Stenmark, 2010; Akhshi et al., 2014). Studies have shown that down-regulating the RAS-driven actin cytoskeleton and phosphatidylinositide 3-kinase/Akt pathway could induce apoptosis and inhibits cell growth, migration and invasion in human BLCA cells (Imai-Sumida et al., 2017).

## 5 Conclusion

In summary, our study identified two related prognostic biomarkers for BLCA: *ANLN* and *ASPM*. High expression of *ANLN* and *ASPM* was associated with poor OS in BLCA. Furthermore, our results showed that *ANLN* and *ASPM* were upregulated in BLCA tissues. We also observed a correlation between the mRNA expression of *ANLN* and *ASPM* in both BLCA and paracancerous tissues. Notably, the increasing multiples of *ANLN* was higher in high-grade BLCA. These results suggest that *ANLN* and *ASPM* genes may be potentially promising targets for BLCA. We continue to explore the interaction mechanism between both *ANLN* and *ASPM* genes *in vitro* and *in vivo* experiments.

## Data Availability

The original contributions presented in the study are included in the article/Supplementary Material, further inquiries can be directed to the corresponding author.
