# Supplementary material for: Screening ANLN and ASPM as bladder urothelial carcinoma-related biomarkers based on weighted gene co-expression network analysis
Source: Front Genet. 2023 Mar 27;14:1107625. doi: 10.3389/fgene.2023.1107625 (PMC10083327; doi:10.3389/fgene.2023.1107625)
Supplement: Supplementary file 1 [file Table1.DOCX]

Table S1. Soft Threshold Screening of GSE32548

| Power | SFT.R.sq | slope | truncated.R.sq | mean.k. | median.k. | max.k. |
| --- | --- | --- | --- | --- | --- | --- |
| 1 | 0.3812 | 0.3849 | 0.5606 | 161.2169 | 1.5517E+02 | 271.5997 |
| 2 | 0.6241 | -0.3784 | 0.7028 | 61.7313 | 5.5165E+01 | 145.4200 |
| 3 | 0.7961 | -0.7335 | 0.8293 | 30.3939 | 2.3697E+01 | 95.5949 |
| 4 | 0.7455 | -0.9400 | 0.7884 | 17.4468 | 1.0851E+01 | 69.5804 |
| 5 | 0.7595 | -1.0154 | 0.8637 | 11.0818 | 5.1319E+00 | 53.8919 |
| 6 | 0.7943 | -1.0587 | 0.9203 | 7.5501 | 2.5688E+00 | 43.4404 |
| 7 | 0.8033 | -1.1005 | 0.9463 | 5.4101 | 1.3893E+00 | 35.9695 |
| 8 | 0.8479 | -1.1477 | 0.9640 | 4.0252 | 7.2979E-01 | 30.3501 |
| 9 | 0.8659 | -1.1524 | 0.9624 | 3.0822 | 4.1841E-01 | 25.9624 |
| 10 | 0.8743 | -1.1590 | 0.9528 | 2.4142 | 2.3886E-01 | 22.4395 |
| 12 | 0.8626 | -1.2196 | 0.9186 | 1.5592 | 7.9201E-02 | 17.1425 |
| 14 | 0.8165 | -1.2912 | 0.8694 | 1.0590 | 3.0398E-02 | 13.3763 |
| 16 | 0.8927 | -1.2289 | 0.9600 | 0.7459 | 1.1831E-02 | 10.5956 |
| 18 | 0.9031 | -1.1892 | 0.9682 | 0.5402 | 4.6347E-03 | 8.4899 |
| 20 | 0.9028 | -1.1930 | 0.9637 | 0.4000 | 1.9056E-03 | 6.8659 |
| 22 | 0.9139 | -1.1901 | 0.9692 | 0.3015 | 8.1964E-04 | 5.5957 |
| 24 | 0.8888 | -1.1879 | 0.9359 | 0.2307 | 3.2826E-04 | 4.5911 |
| 26 | 0.9278 | -1.1484 | 0.9748 | 0.1789 | 1.4089E-04 | 3.7892 |
| 28 | 0.9564 | -1.1310 | 0.9846 | 0.1403 | 5.9423E-05 | 3.1439 |
| 30 | 0.9557 | -1.1362 | 0.9725 | 0.1111 | 2.5317E-05 | 2.6210 |


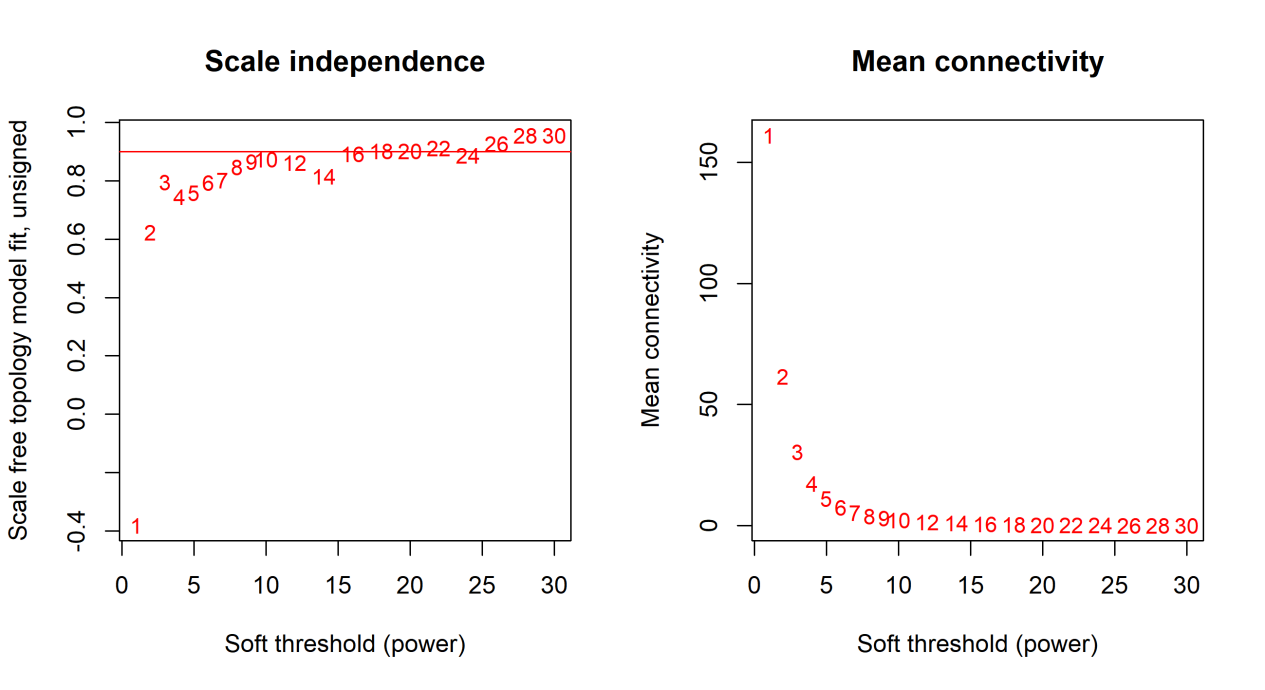


**A**

**B**


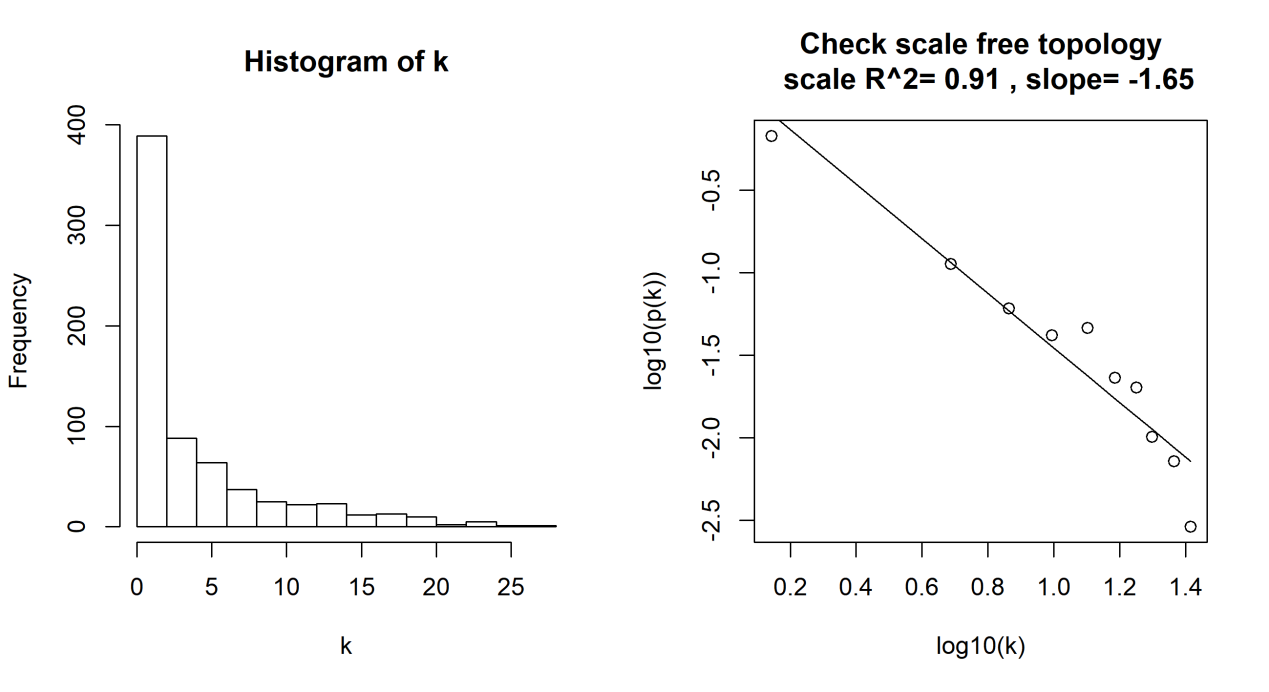


**D**

**C**

Figure S1. Soft Threshold Screening for GSE32548. (A) The correlation coefficient of the scale free topology fitting index (SFT.R.sq, *R*^2^) corresponding to different soft thresholds. (B) The average connection degree of genes corresponding to different soft thresholds. (C) For the selected soft threshold *β* = 9, the histogram of the connectivity of each node. (D) The scatter plot of log(p(k)) and log(k). The linear regression results show that the correlation coefficient is 0.91, It conforms to the scale-free network characteristics.


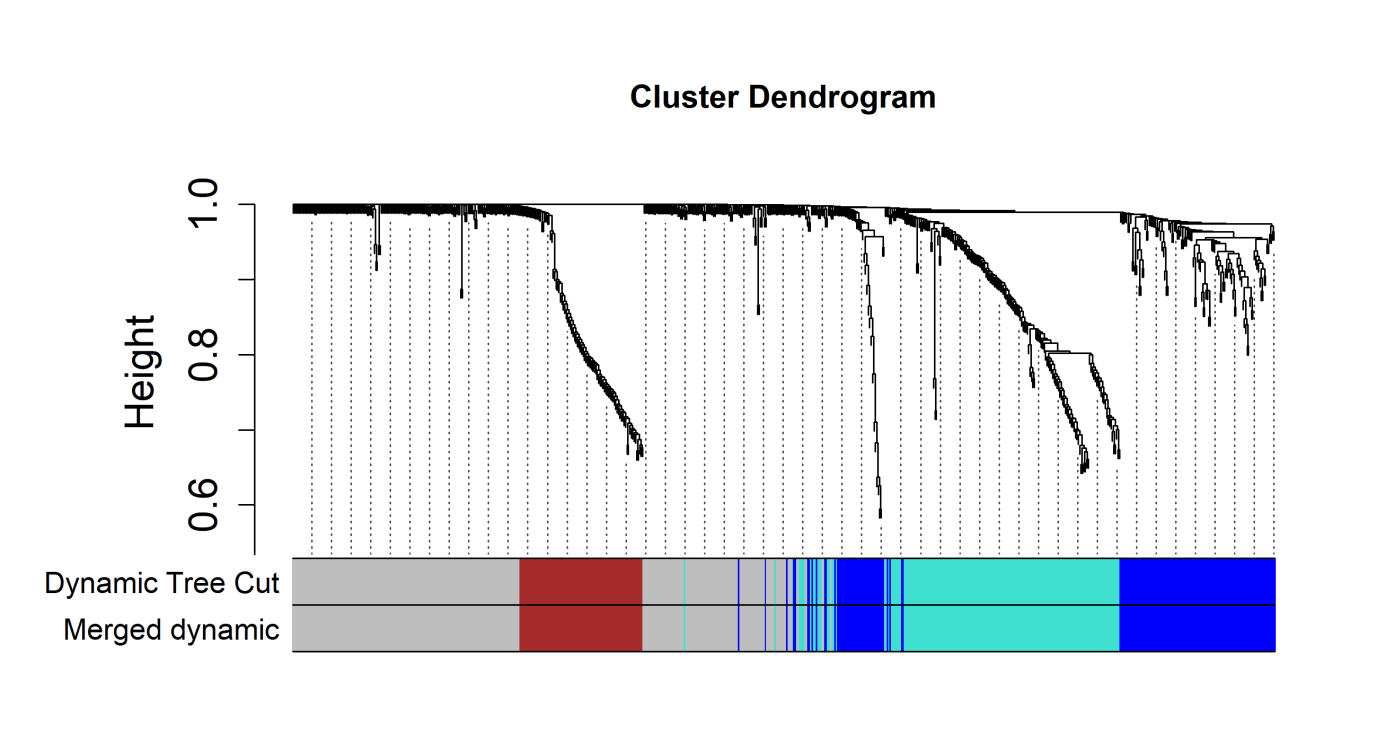


Figure S2. Classification of GSE32548 gene clustering trees and merge of high similarity high modules by dynamic hybrid cut.
